# Evaluation of transcriptionally regulated genes identifies NCOR1 in hormone receptor negative breast tumors and lung adenocarcinomas as a potential tumor suppressor gene

**DOI:** 10.1371/journal.pone.0207776

**Published:** 2018-11-28

**Authors:** María del Mar Noblejas-López, Sara Morcillo-García, Cristina Nieto-Jiménez, Miriam Nuncia-Cantarero, Balázs Győrffy, Eva M. Galan-Moya, Atanasio Pandiella, Alberto Ocaña

**Affiliations:** 1 Translational Research Unit, Albacete University Hospital, and CIBERONC, Albacete, Spain; 2 Centro Regional de Investigaciones Biomédicas, Universidad de Castilla-La Mancha, Albacete, Spain; 3 Semmelweis University 2nd Dept. of Pediatrics, Budapest, Hungary; 4 MTA TTK Lendület Cancer Biomarker Research Group, Institute of Enzymology, Budapest, Hungary; 5 Cancer Research Center, CSIC-IBSAL and CIBERONC, Salamanca, Spain; Florida International University, UNITED STATES

## Abstract

Regulation of transcription is a key process in cellular homeostasis. It depends on regulators that either repress or stimulate the transcription of genes, therefore controlling different biological functions. The Nuclear Receptor Corepressor 1 (NCOR1) is one of those co-repressors that regulate the transcription by facilitating the recruitment of HDAC1, 2, 3, 4, 5 and 7. In our article, by using an *in silico* approach, we evaluate the mutational status of NCOR1 in breast and lung tumors. We identified that NORC1 is mutated in more than 3% of breast tumors and lung adenocarcinomas and linked this fact with detrimental outcome in some subtypes, particularly in those that are hormone receptor negative. In addition to these findings, as mutations in this gene are deleterious, we confirmed that high levels of this gene were linked with good prognosis in the same tumor subtypes. Findings in the same direction were identified in lung adenocarcinomas, with mutations associated with detrimental prognosis and high expression with better outcome. In conclusion, hereby we describe the presence and prognostic role of mutations in the NCOR1 gene in hormone receptor negative breast and lung adenocarcinomas, and we also confirm that NCOR1 is a tumor suppressor gene. Further studies should be performed to explore therapeutic mechanisms to restore its function.

## Introduction

Regulation of transcription is a key process in cellular homeostasis [1, 2]. It depends on regulators that either repress or stimulate the transcription of genes, therefore controlling different biological functions [1, 2]. In this context, modifications of the transcription process have been associated with human disorders, such as neurological or inflammatory diseases or cancer, among others [1, 2].

Activation of transcription is regulated at different levels, and epigenetic mechanisms play a central role [1, 3]. Notably, epigenetic modifications on histones can impact on the transcription of different genes, and its deregulation has been involved in the initiation and progression of tumors [1].

Acetylation of histones is mediated by enzymes like histone acetyltransferases and deacetylase (HDAC), that can modify the conformation of the chromatin and therefore affect the transcriptional activity [2].

The function of these enzymes depends, in many occasions, on the presence of co-repressors, which can facilitate or repress the transcription of several genes [4]. The Nuclear Receptor Corepressor 1 (NCOR1) is one of those co-repressors that regulates the transcription by facilitating the recruitment of HDAC1, 2, 3, 4, 5 and 7 [5]. Among all, HDAC3 is probably the principal responsible of its activity, as the catalytic activity of this enzyme requires interaction with NCOR [3, 6].

NCOR1 plays a central role in human biology, being involved in many process including lipid metabolisms, cell fate, glucose homeostasis or neural stem cells [5]. Indeed, germline mutations of NCOR produce embryonic lethality [7]. NCOR1 has also been involved in cancer [5, 8, 9]. Mutations or deletions of this gene have been described in several solid tumors, such as colorectal cancer, bladder cancer or hepatocarcinomas [5, 8, 9]. Indeed, it has been considered as a tumor suppressor gene as reduced levels of the gene promote tumor proliferation and invasion [5].

In our article, we evaluate the mutational status of NCOR1 in breast and lung tumors, focusing on genes involved in transcriptional regulation. We identified that NCOR1 is mutated in more than 3% of breast tumors and lung adenocarcinomas and linked with detrimental outcome in some subtypes. In addition to these findings, as mutations at this gene are deleterious, we confirmed that high levels of this gene were linked with good prognosis in those tumor types.

## Material and methods

### Identification of high-frequency mutated genes

Breast Cancer METABRIC database, contained in cBioportal (http://www.cbioportal.org), includes genomic information from 2509 breast cancer samples [10]. Then, we selected the 772 samples from patients diagnosed with Invasive Breast Carcinoma (S1 Table). We studied the gene mutation profile and selected those genes presented in more than 2% of the patients. The frequency of mutation was independently confirmed for all the four different breast cancer subtypes using TCGA database (n = 818).

NCOR1 frequency of mutation in lung adenocarcinoma and squamous cell lung carcinoma (n = 230 and n = 178, respectively) was extracted from TCGA database contained at cBioPortal (http://www.cbioportal.org).

### Functional analysis

The selected mutated genes were analyzed using the biological functional enrichment analyses tool Enrichr (http://www.amp.pharm.mssm.edu/Enrichr/). An adjusted *p*-value <0.05 was used to select the enriched gene-sets. This tool was also used to evaluate genes present in the mutated NCOR1 signature.

### Outcome analyses

The genotype-2-Outcome online tool (http://www.g-2-o.com) was used to evaluate the relationship between the presence of mutated NCOR1 and patient clinical outcome through the development of a NCOR1 expression signature associated with all NCOR1 mutations [11]. This includes not only patients with NCOR1 mutation, but all patients who have a gene expression signature similar to those with an NCOR1 mutation. This publicly available database allowed assessment of clinical outcome (overall survival (OS)) for all the four breast cancer subtypes (All, Triple Negative Breast Cancer, Luminal A, Luminal B and HER2+) and (relapse free survival (RFS)) for lung adenocarcinomas. In the analysis, the expression of genes related to NCOR1 mutation was computed for each sample. Then, the upper quartile (25% with higher expression) or the median across all patients was used to define two cohorts, and each patient was assigned to those with higher expression or to those with lower expression, based on the threshold defined for the upper quartile or the median value. The two cohorts were compared using a Cox regression analysis.

KM Plotter Online Tool (http://www.kmplot.com) was used to investigate the relationship between the NCOR1 expression and patient clinical outcome. This database allowed assessment of overall survival (OS) and relapse-free survival (RFS) for all subtypes, basal-like, luminal A, luminal B, HER2+ and lung adenocarcinoma.

Patients were separated according to upper quartile or best cutoff values. For the latter, all possible cutoff values between the lower and the upper quartiles are analyzed, and the one with the lowest p value was used as a cutoff to separate the two patient cohorts. Patients above the threshold were considered to have a “high” expression while patients below the threshold were defined as those with “low” expression.

### Evaluation of NCOR1 mutations

The information contained at cBioportal (http://www.cbioportal.org) was used to identify NCOR1 mutations. The effect of each mutation on NCOR1 activity was investigated in SIFT (http://sift.bii.a-star.edu.sg/) and PolyPhen-2 (http://genetics.bwh.harvard.edu/pph2/) databases.

### Assessment of NCOR1 association with therapy

We have searched GEO to identify breast cancer transcriptomic datasets with published treatment and follow-up data as described previously [12, 13] to link gene expression levels to patient’s response to the received therapy (Chemotherapy, Endocrine-therapy and Anti-HER2 therapy). Response was determined using relapse-free survival at five years: patients with a relapse before five years were designated as non-responders, those without a relapse before five years were designated as responders. Patients censored before five years follow-up were excluded from the analysis. We performed Mann-Whitney analysis comparing responder and non-responder patients in order to study the association of NCOR1 with therapy response. Molecular subtypes were determined as described previously [14].

## Results

### Identification of mutations in transcriptional-regulated genes

**Fig 1:**
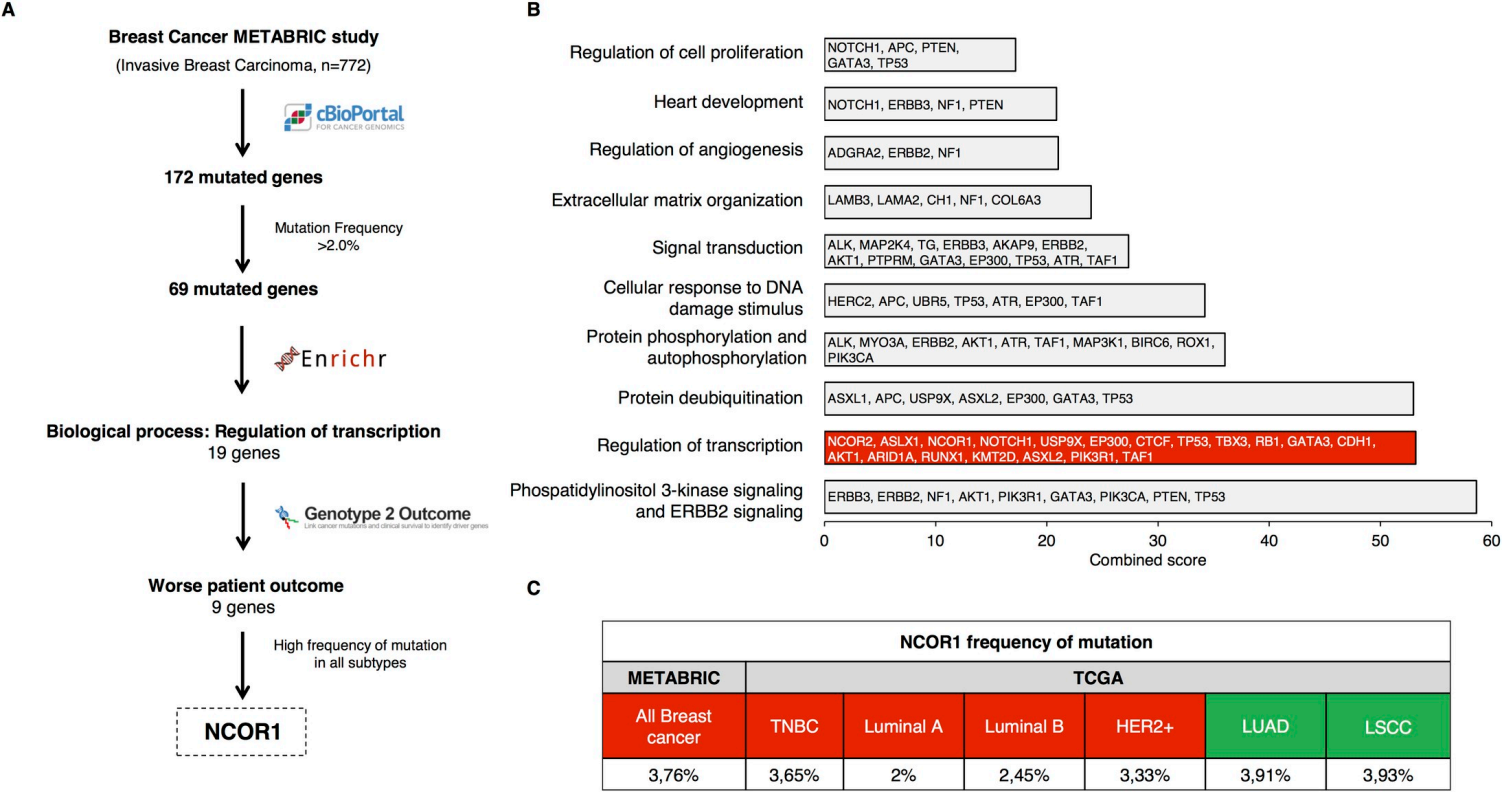
Whole genome mutation profiling and identification of regulation of transcription as an altered function in breast cancer. A. Flow chart of the study. B. Functional analyses of the selected 69 mutated genes (Mutation frequency > 2.0%). C. NCOR1 frequency of mutation for the overall of the breast cancer cases (All Breast Cancer), according to METABRIC (n = 2059), was confronted with data from the TCGA database (n = 818), which allows the estimation of the frequency of mutation for each breast cancer subtype individually. This database was also used to extract the frequency of NCOR1 mutations in Lung Adenocarcinoma (LUAD) (n = 230) and Lung Squamous Cells Carcinoma (LSCC) patients (n = 178).

We used public information from the METABRIC study (n = 772) to study genes mutated in breast cancer patients (Fig 1A). We found a total of 172 altered genes, of which 69 were mutated in more than 2% of the patients. Next, we investigated their function using gene set enrichment analyses and we focused on those involved in the regulation of transcription (Fig 1B). In addition, taking advantage of the online tool “genome 2 outcome”, which links gene mutations with a transcriptomic signature associated with that mutation, we selected those genes that predicted detrimental prognosis. With this approach, we found 9 mutated genes associated with worse patient outcome (Fig 1A and S2 Table). Some of these genes have been deeply explored in breast cancer, like TP53, RB1 or PiK3R1. However, others have been less studied, like TAF1, KTM2D, RUNX1, EP300 or NCOR1 and NCOR2. We put our attention on NCOR1, as it was the only one associated with detrimental outcome in a significant manner in all breast cancer subtypes. Besides, little is known about its role in hormone receptor-negative breast tumors. Data obtained from the METABRIC study was corroborated with data extracted from the TCGA database. As shown, NCOR1 was mutated in 3.76% of all breast tumors, and in 3.65% of basal-like, 2% of Luminal A, 2.45% of Luminal B, and 3.33% of HER2 positive (Fig 1C).

### Presence of NCOR1 mutations predict detrimental prognosis in triple negative, HER2 positive and luminal B tumors

**Fig 2:**
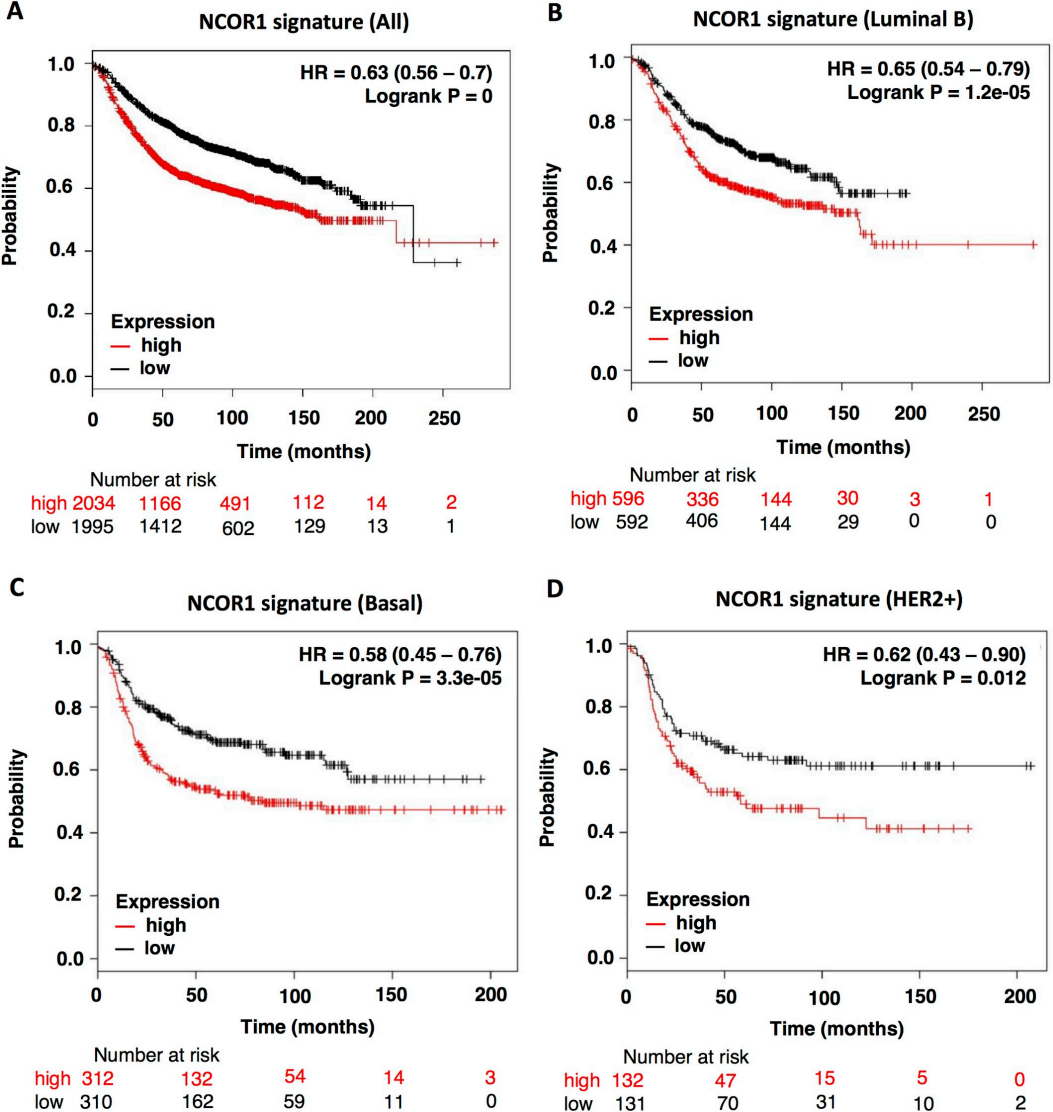
NCOR1 mutation-associated signature correlates with patients’ poor outcome. OS plots showing the association between survival and NCOR1-mutation-related signature in all breast cancers (A), luminal B (B), basal (C) and HER2+ breast tumors (D) were obtained using Genotype-2-Outcome. This tool found 58, 36, 11 and 7 patients carrying NCOR1 mutations in all breast cancer tumours, luminal B, basal and HER2 positive tumours, respectively. The patients were separated in “high or low” expression groups according to the median value of the mean expression of NCOR1-mutation associated genes.

Using the NCOR1 mutational-transcriptomic signature as a read out of the mutation, we explored the association of this signature with prognosis, overall survival (OS) specifically. Mutations at NCOR1 were associated with detrimental outcome in all breast tumors (HR:0.63, CI: 0.56–0.7; log rank p = 0), luminal B (HR:0.65, CI: 0.54–0.79; log rank p = 1.2e-05), basal (HR:0.58, CI: 0.45–0.76; log rank p = 3.3e-05) and HER2 positive tumors (HR:0.62, CI: 0.43–0.90; log rank p = 0.012) (Fig 2A, 2B, 2C and 2D, respectively). The low prevalence of mutations in the luminal A subtype limited the analyses between mutation and clinical outcome in this subtype.

### Functional analysis of the NCOR1 mutational-transcriptomic signature

**Fig 3:**
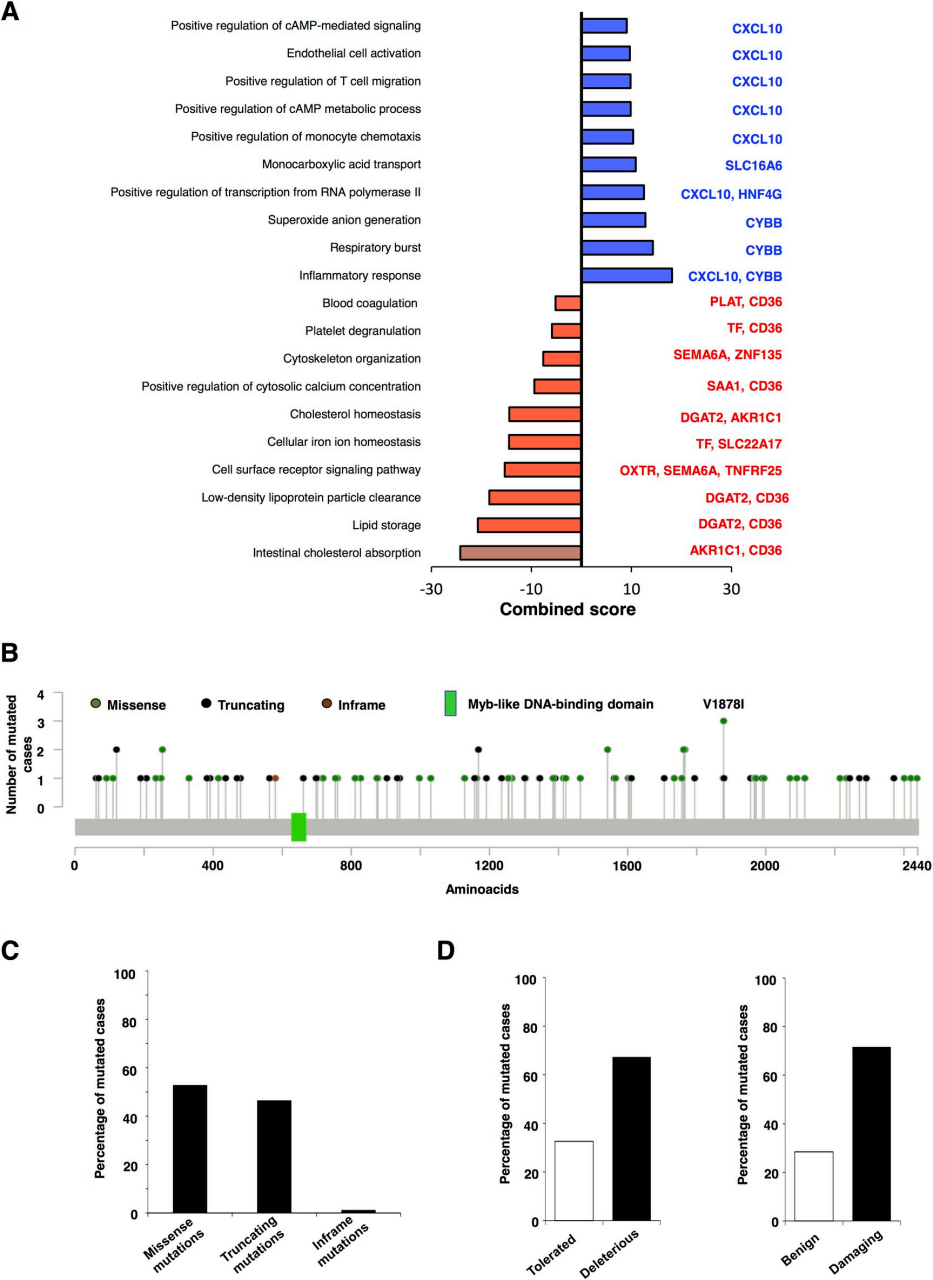
Functional analysis of the NCOR1 signature. A. Mutated NCOR1-associated up (blue) and downregulated (red) biological process. NCOR1-associated genes within each function are indicated. B. Schema of NCOR1 mutations. C. Percentage of frequency of Missense, Truncating and Inframe mutations. D. Percentage of Tolerated versus Deleterious and Benign versus Damaging mutations.

Next, we explored which genes were included in the NCOR1 mutational signature and their biological function. Genes down-regulated in NCOR1-mutated tumors include those involved in lipid storage like DGAT2 and CD36, or cell surface receptor signaling including genes like OXTR, SEMA6A or TNFRF25. Up-regulated genes comprised CXCL10, CYBB, SLC16A6 or HNF4G (Fig 3A).

### NCOR1 mutations and loss of function

As mutations of this gene have an impact on patient outcome, we aimed to analyze the biological role of these mutations on the expression of the protein and its function. To do so, we used the cancer genomics database cBioportal to identify *NCOR1* mutations in each METABRIC patient (Fig 3B). Missense and truncating mutations were the most frequent mutations identified with only one case of inframe mutations (Fig 3C). The functional impact of all these different mutations, evaluated with two different databases (SIFT and PolyPhen-2), is displayed in Fig 3D. As shown, most NCOR1 mutations are deleterious or damaging, therefore affecting the functional role of the protein.

### High NCOR1 transcriptomic levels are associated with good prognosis

**Fig 4:**
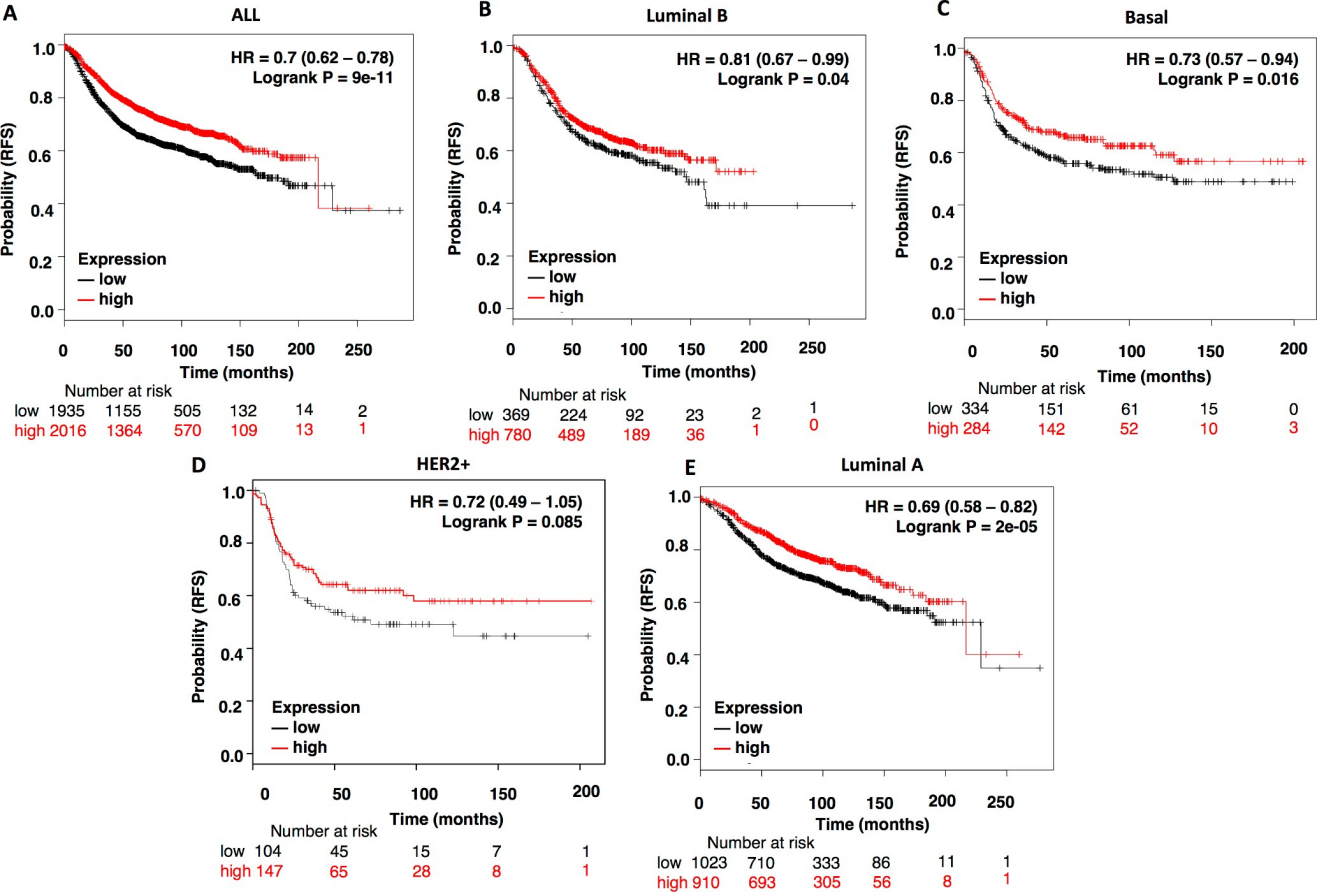
High NCOR1 expression levels correlates with better patients’ prognosis. Kaplan-Meier survival plots showing the association of NCOR1 expression levels with patients’ relapse free survival (RFS) for all breast cancers (n = 3951) (A), luminal B (n = 1149) (B), basal (n = 618) (C), HER2+ (n = 251) (D) and Luminal A (n = 1933) (E) breast tumors.

If lack of activity of NCOR1 due to mutations is associated with detrimental outcome, elevated levels of the gene would be linked with good prognosis. To explore this idea, we evaluated NCOR1 at a transcriptomic level in relation with outcome in the different breast cancer subtypes. As can be seen in Fig 4, high expression of NCOR1 is associated with better relapse free survival in all breast cancer subtypes, including the luminal A subtype: all breast tumors (HR: 0.7, CI: 0.62–0.78; log rank p = 9e-11); luminal B (HR: 0.81, CI: 0.67–0.99; log rank p = 0.04); basal (HR: 0.73, CI: 0.57–0.94; log rank p = 0.016); HER2 (HR:0.72, CI: 0.49–1.05; log rank p = 0.085) and luminal A (HR:0.69, CI: 0.58–0.82; log rank p = 2e-05) (Fig 4A–4E).

To investigate the possibility that NCOR1 levels could be associated with response to therapy, we correlated gene expression with clinical outcome based on the response to a selected treatment. Expression levels of NCOR1 did not discriminate responders to anti-HER2 therapy (n = 50) or chemotherapy (n = 476) in neither the HER2+ER- or triple negative subtypes (S3 Table). However, as it has been previously described (see discussion), expression of this gene was able to predict response to anti-hormonal therapy (n = 907).

### Presence of NCOR1 mutations, transcriptomic expression and association with survival in lung cancers

**Fig 5:**
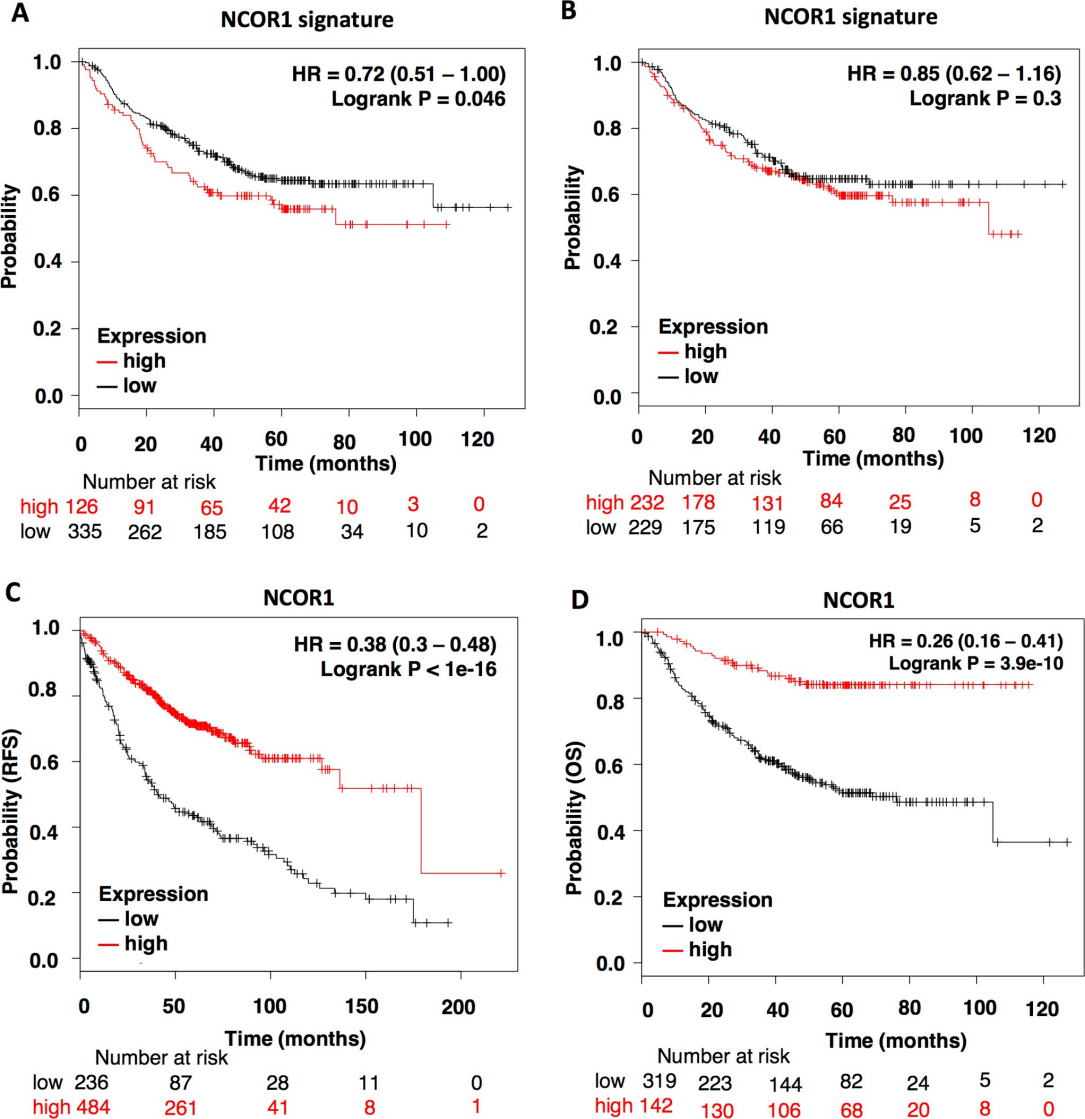
NCOR1 expression profile also predict outcome in Lung Adenocarcinoma patients (LUAD). A-B. Survival plots showing the correlation of NCOR1-mutation related signature with RFS. The patients were separated in “high or low” using upper quartile expression (A) or median expression as the cutoff value (B). C-D. Kaplan-Meier survival plots showing the association of NCOR1 expression levels with patients RFS (n = 720) and OS (n = 461).

To explore the option that NCOR1 also have a role in other cancer types, we explored if mutations at this gene were linked with detrimental prognosis in lung cancer. Although no association was observed for all the groups, a significant detrimental outcome (RFS) was identified for the subgroup of lung adenocarcinomas (LUAD), particularly for the upper-quartile group (HR: 0.72, CI: 0.51–1.00; log rank p = 0.046) (Fig 5A), compared with median cut-off (HR: 0.85, CI: 0.62–1.16; log rank p = 0.3) (Fig 5B). Of note, mutations in LUAD was reported in 3.91% of tumors (Fig 1C). Similarly, to breast cancer, we observed that high expression of the NCOR1 gene was associated with better overall survival and relapse free survival (HR: 0.38, CI: 0.3–0.48; log rank p<1e-16; HR: 0.26, CI: 0.16–0.41; log rank p = 3.9e-10; respectively) (Fig 5C and 5D), suggesting that this gene could behave as a tumor suppressor also in this tumor subtype.

## Discussion

In the present article, we explore mutations associated with genes involved in transcriptional regulation that are linked with detrimental outcome. Among all the identified genes, we focused on NCOR1, as this gene has not been described previously in hormone receptor negative breast tumors.

Deregulation of genes within transcription regulation has been involved in human pathologies, including tumor initiation and progression [5,9]. In our study, we identify different mutated genes within this function including some previously described and other that are more novel. We focused on NCOR1 as this gene was associated with detrimental outcome in all breast subtypes, and its association with survival in hormone receptor negative tumors has not been evaluated before.

Some studies associate loss of expression of NCOR1 with cancer propagation and proliferation. Indeed, NCOR represses the expression of prometastatic genes like CXCR4, COX2, CCR6 and CCR1 [15,16]. NCOR1 has been deeply studied in estrogen receptor positive breast tumors. Loss of expression of NCOR1 has been associated with resistance to hormone therapy, particularly tamoxifen as NCOR1 is a key corepressor for ERα [17,18]. Of note, this data is also in line with our observation linking the presence of the gene with resistance to tamoxifen or aromatase inhibitors. However, the role of NCOR1 mutations, particularly in tumors that do not express the estrogen receptor has not been explored in the mentioned studies.

Mutations in NCOR1 have been also described in other solid tumors, like colorectal cancer or bladder cancer [8, 9]. In most occasions, these mutations were associated with a loss of function of the protein impairing their tumor suppressor capabilities [5, 8]. In our article, we found that NCOR1 mutations in breast cancer are mainly missense and truncating, and produce a deleterious or damaging effect, leading to a nonfunctional protein. The lack of function of this protein is therefore associated with the poor prognosis observed in those patients harboring the mutation. By contrast, we observed that tumors with elevated transcriptomic levels of NCOR1 were linked with good prognosis as the protein levels are higher. Our findings were also confirmed in lung adenocarcinomas, in which no data has been previously described.

A relevant observation was the fact that the expression of the gene was not associated with clinical outcome based on the predicted response to anti-HER2 therapies or chemotherapies, contrary to those treated with hormonotherapy. This confirms the role of NCOR1 as a tumor suppressor gene in the estrogen receptor negative tumors.

Finally, it should be mentioned that the low number of mutations in luminal A tumors limits their evaluation in relation with prognosis. We could anticipate that if a dysfunctional protein does exist, then, resistance to hormonotherapy could be present.

In conclusion, in the present work we describe the presence and prognostic role of mutations at NCOR1 gene in hormone receptor negative breast and lung adenocarcinomas and we also confirm that NCOR1 is a tumor suppressor gene. Further studies should be performed to explore therapeutic mechanisms to restore its function.

## Supporting information

S1 Table Clinical data from the invasive breast carcinoma patients contained in the METABRIC study. (PDF)

S2 Table Selected mutated genes association with patients’ prognosis. (PDF)

S3 Table Association of NCOR1 expression with therapy response. Relapse-free survival at five years was used to assign patients to responder (no relapse before 5 years) and non-responder (relapsed before five years) cohorts. Only endocrine therapy (n = 907) reaches high significance while the correlation is negligible for anti-HER2 therapy (n = 50) and chemotherapy (n = 476).
(PDF)

## References

[1] Arrowsmith CH, Bountra C, Fish PV, Lee K, Schapira M. Epigenetic protein families: a new frontier for drug discovery. Nat Rev Drug Discov 2012; 11: 384–400. 10.1038/nrd3674 22498752 10.1038/nrd3674

[2] Grunstein M. Histone acetylation in chromatin structure and transcription. Nature 1997; 389: 349–352. 10.1038/38664 9311776 10.1038/38664

[3] Bhaskara S, Knutson SK, Jiang G, Chandrasekharan MB, Wilson A J, Zheng S et al Hdac3 is essential for the maintenance of chromatin structure and genome stability. Cancer Cell 2010; 18: 436–447. 10.1016/j.ccr.2010.10.022 21075309 10.1016/j.ccr.2010.10.022 PMC3004468

[4] Perissi V, Aggarwal A, Glass CK, Rose DW, Rosenfeld MG. A corepressor/coactivator exchange complex required for transcriptional activation by nuclear receptors and other regulated transcription factors. Cell 2004; 116: 511–526. 14980219 10.1016/s0092-8674(04)00133-3

[5] Martinez-Iglesias O, Alonso-Merino E, Aranda A. Tumor suppressive actions of the nuclear receptor corepressor 1. Pharmacol Res 2016; 108: 75–79. 10.1016/j.phrs.2016.04.027 27149915 10.1016/j.phrs.2016.04.027

[6] Guenther MG, Barak O, Lazar MA. The SMRT and N-CoR corepressors are activating cofactors for histone deacetylase 3. Mol Cell Biol 2001; 21: 6091–6101. 10.1128/MCB.21.18.6091-6101.2001 11509652 10.1128/MCB.21.18.6091-6101.2001 PMC87326

[7] Jepsen K, Hermanson O, Onami TM, Gleiberman AS, Lunyak V, McEvilly RJ et al Combinatorial roles of the nuclear receptor corepressor in transcription and development. Cell 2000; 102: 753–763. 11030619 10.1016/s0092-8674(00)00064-7

[8] Ivanov I, Lo KC, Hawthorn L, Cowell JK, Ionov Y. Identifying candidate colon cancer tumor suppressor genes using inhibition of nonsense-mediated mRNA decay in colon cancer cells. Oncogene 2007; 26: 2873–2884. 10.1038/sj.onc.1210098 17086209 10.1038/sj.onc.1210098

[9] Gui Y, Guo G, Huang Y, Hu X, Tang A, Gao S et al Frequent mutations of chromatin remodeling genes in transitional cell carcinoma of the bladder. Nat Genet 2011; 43: 875–878. 10.1038/ng.907 21822268 10.1038/ng.907 PMC5373841

[10] Pereira B, Chin SF, Rueda OM, Vollan HK, Provenzano E, Bardwell HA et al The somatic mutation profiles of 2,433 breast cancers refines their genomic and transcriptomic landscapes. Nat Commun. 2016;7:11479 10.1038/ncomms11479 27161491 10.1038/ncomms11479 PMC4866047

[11] Pongor L, Kormos M, Hatzis C, Pusztai L, Szabó A, Győrffy B et al A genome-wide approach to link genotype to clinical outcome by utilizing next generation sequencing and gene chip data of 6,697 breast cancer patients. Genome Med 2015; 7: 104 10.1186/s13073-015-0228-1 26474971 10.1186/s13073-015-0228-1 PMC4609150

[12] Mihaly Z, Kormos M, Lanczky A, Dank M, Budczies J, Szász MA et al A meta-analysis of gene expression-based biomarkers predicting outcome after tamoxifen treatment in breast cancer. Breast Cancer Res Treat 2013; 140: 219–232. 10.1007/s10549-013-2622-y 23836010 10.1007/s10549-013-2622-y

[13] Menyhart O, Budczies J, Munkacsy G, Esteva FJ, Szabó A, Miquel TP et al DUSP4 is associated with increased resistance against anti-HER2 therapy in breast cancer. Oncotarget 2017; 8: 77207–77218. doi: 10.18632/oncotarget.20430 29100381 10.18632/oncotarget.20430 PMC5652774

[14] Gyorffy B, Lanczky A, Eklund AC, Denkert C, Budczies J, Li Q et al An online survival analysis tool to rapidly assess the effect of 22,277 genes on breast cancer prognosis using microarray data of 1,809 patients. Breast Cancer Res Treat 2010; 123: 725–731. 10.1007/s10549-009-0674-9 20020197 10.1007/s10549-009-0674-9

[15] Martinez-Iglesias O, Olmeda D, Alonso-Merino E, Gómez-Rey S, González-López AM, Luengo E et al The nuclear corepressor 1 and the thyroid hormone receptor beta suppress breast tumor lymphangiogenesis. Oncotarget 2016; 7: 78971–78984. doi: 10.18632/oncotarget.12978 27806339 10.18632/oncotarget.12978 PMC5346691

[16] Martinez-Iglesias OA, Alonso-Merino E, Gomez-Rey S, Velasco-Martín JP, Martín Orozco R, Luengo E et al Autoregulatory loop of nuclear corepressor 1 expression controls invasion, tumor growth, and metastasis. Proc Natl Acad Sci U S A 2016; 113: E328–337. 10.1073/pnas.1520469113 26729869 10.1073/pnas.1520469113 PMC4725484

[17] Lu R, Hu X, Zhou J, Sun J, Zhu AZ, Xu X et al COPS5 amplification and overexpression confers tamoxifen-resistance in ERalpha-positive breast cancer by degradation of NCoR. Nat Commun 2016; 7: 12044 10.1038/ncomms12044 27375289 10.1038/ncomms12044 PMC4932188

[18] Cutrupi S, Reineri S, Panetto A, Grosso E, Caizzi L, Ricci L et al Targeting of the adaptor protein Tab2 as a novel approach to revert tamoxifen resistance in breast cancer cells. Oncogene 2012; 31: 4353–4361 10.1038/onc.2011.627 22249258 10.1038/onc.2011.627

