# Supplementary material for: Evaluation of transcriptionally regulated genes identifies NCOR1 in hormone receptor negative breast tumors and lung adenocarcinomas as a potential tumor suppressor gene
Source: PLoS One. 2018 Nov 28;13(11):e0207776. doi: 10.1371/journal.pone.0207776 (PMC6261593; doi:10.1371/journal.pone.0207776)
Supplement: S1 Table — (PDF) [file pone.0207776.s001.pdf]

BRCA_Invasive breast carcinoma_METABRIC_Clinical Data

	Condition	Number of patients	% of patients
**Histologic Grade**	NA	553	71,63
	3	64	8,29
	2	93	12,05
	1	62	8,03
**Chemotherapy**	NA	529	68,52
	NO	205	26,55
	YES	38	4,92
**HER2 Status**	NA	529	68,52
	Positive	223	28,89
	Negative	20	2,59
**Age at Diagnosis**	NA	529	68,52
	>70	54	6,99
	50-70	133	17,23
	30-50	53	6,87
	20-30	1	0,13
**Overall Survival Status**	NA	529	68,52
	>200	62	8,03
	100<x<200	103	13,34
	50<x<100	43	5,57
	<50	32	4,15
**Pam50 + Claudin-low subtype**	NA	529	68,52
	LumA	94	12,18
	LumB	46	5,96
	claudin-low	50	6,48
	Basal	21	2,72
	Normal	19	2,46
	Her2	12	1,55
	NC	1	0,13
**Tumor Stage**	NA	574	74,35
	4	2	0,26
	3	7	0,91
	2	79	10,23
	1	102	13,21
	0	8	1,04

Supplementary Table 1
