# Supplementary material for: Evaluation of transcriptionally regulated genes identifies NCOR1 in hormone receptor negative breast tumors and lung adenocarcinomas as a potential tumor suppressor gene
Source: PLoS One. 2018 Nov 28;13(11):e0207776. doi: 10.1371/journal.pone.0207776 (PMC6261593; doi:10.1371/journal.pone.0207776)
Supplement: S2 Table — (PDF) [file pone.0207776.s002.pdf]

	All		TNBC		Luminal A		Luminal B		HER2+	
	HR	P-valor	HR	P-valor	HR	P-valor	HR	P-valor	HR	P-valor
**NCOR2**	0,83 (0,74 - 0,92)	0,00066	0,97 (0,76 - 1,25)	0,84	Not possible		1,2 (1,0 - 1,5)	0,05	Not possible	
**ASLX1**	0,96 (0,86 - 1,07)	0,42	1,20 (0,94 - 1,55)	0,15	Not possible		0,98 (0,81 - 1,19)	0,85	Not possible	
**NCOR1**	0,63 (0,56 - 0,7)	0	0,58 (0,45 - 0,76)	3,30E-05	Not possible		0,65 (0,54 - 0,79)	1,20E-05	0,62 (0,43 - 0,9)	0,012
**NOTCH1**	0,91 (0,82 - 1,01)	0,088	0,58 (0,45 - 0,75)	2,60E-05	Not possible		1,3 (1,1 - 1,6)	0,0025	Not possible	
**USP9X**	Not possible		0,73 (0,56 - 0,93)	0,012	0,53 (0,45 - 0,64)	7,40E-13	No possible		0,61 (0,42 - 0,88)	0,0083
**EP300**	0,66 (0,59 - 0,74)	4,70E-14	0,89 (0,70 - 1,15)	0,38	Not possible		0,94 (0,77 - 1,13)	0,49	0,89 (0,61 - 1,29)	0,54
**CTCF**	Not possible		Not possible		Not possible		1,06 (0,88 - 1,28)	0,54	Not possible	
**TP53**	0,55 (0,49 - 0,61)	0	0,86 (0,67 - 1,10)	0,22	0,47 (0,39 - 0,56)	0	0,82 (0,68 - 1)	0,045	0,62 (0,42 - 0,9)	0,011
**TBX3**	1,3 (1,2 - 1,5)	1,20E-06	Not possible		Not possible		1,17 (0,97 - 1,42)	0,099	Not possible	
**RB1**	0,72 (0,64 - 0,80)	1,90E-09	1,01 (0,79 - 1,30)	0,95	Not possible		1,06 (0,88 - 1,28)	0,55	Not possible	
**CDH1**	1,4 (1,2 - 1,5)	3,90E-09	0,62 (0,48 - 0,80)	0,00017	0,96 (0,81 - 1,139	0,62	1,3 (1,1 - 1,6)	0,0026	Not possible	
**AKT1**	1,5 (1,3 - 1,7)	9,70E-13	0,96 (0,75 - 1,24)	0,76	Not possible		0,95 (0,78 - 1,14)	0,56	Not possible	
**GATA3**	1,6 (1,4 - 1,8)	0	Not possible		Not possible		1,3 (1,0 - 1,5)	0,015	Not possible	
**ARID1A**	1,01 (0,91 - 1,13)	0,83	0,78 (0,60 - 1,00)	0,046	Not possible		0,93 (0,77 - 1,12)	0,44	Not possible	
**RUNX1**	0,78 (0,70 - 0,87)	6,60E-06	0,61 (0,48 - 0,79)	0,00015	Not possible		1,4 (1,1 - 1,7)	0,0013	Not possible	
**KTM2D**	0,62 (0,56 - 0,69)	0	0,58 (0,45 - 0,74)	1,90E-05	0,73 (0,62 - 0,87)	4,00E-04	0,66 (0,55 - 0,80)	1,90E-05	0,75 (0,52 - 1,09)	0,13
**ASXL2**	1,2 (1,0 - 1,3)	0,0065	0,66 (0,51 - 0,85)	0,0012	Not possible		1,5 (1,2 - 1,8)	2,90E-05	Not possible	
**PIK3R1**	0,64 (0,57 - 0,71)	6,70E-16	Not possible		Not possible		0,69 (0,57 - 0,83)	0,00011	Not possible	
**TAF1**	0,69 (0,61 - 0,76)	8,80E-12	0,75 (0,58 - 0,96)	0,023	Not possible		1,07 (0,89 - 1,29)	0,48	0,76 (0,53 - 1,11)	0,16

Supplementary Table 2
