# Supplementary material for: Evaluation of transcriptionally regulated genes identifies NCOR1 in hormone receptor negative breast tumors and lung adenocarcinomas as a potential tumor suppressor gene
Source: PLoS One. 2018 Nov 28;13(11):e0207776. doi: 10.1371/journal.pone.0207776 (PMC6261593; doi:10.1371/journal.pone.0207776)
Supplement: S3 Table — Relapse-free survival at five years was used to assign patients to responder (no relapse before 5 years) and non-responder (relapsed before five years) cohorts. Only endocrine therapy (n = 907) reaches high significance while the correlation is negligible for anti-HER2 therapy (n = 50) and chemotherapy (n = 476). (PDF) [file pone.0207776.s003.pdf]

	NCOR1						
	Relapse-free survival						
	Chemotherapy		Endocrine therapy			Anti-HER2 therapy	
	All	TNBC	All	Luminal A	Luminal B	All	HER2+
Mann-Whitney test	24543,5	3180,5	41200,5	17893,5	4286,5	301,5	60
Mann-Whitney test p- value	0,016	0,56	6,70E-05	0,0011	0,046	0,96	0,51
Fold change	1,1	1,1	1,2	1,1	1,2	0,98	1,1

Supplementary Table 3
